# Lamivudine/dolutegravir dual therapy in HIV-infected, virologically suppressed patients

**DOI:** 10.1186/s12879-017-2311-2

**Published:** 2017-03-16

**Authors:** Franco Maggiolo, Roberto Gulminetti, Layla Pagnucco, Margherita Digaetano, Simone Benatti, Daniela Valenti, Annapaola Callegaro, Diego Ripamonti, Cristina Mussini

**Affiliations:** 1 Division of Infectious Diseases, ASST Papa Giovanni XXIII, Piazza OMS 1, 24127 Bergamo, Italy; 2 Division of Infectious Diseases, Fondazione IRCCS Policlinico San Matteo, Pavia, Italy; 3 Division of Infectious Diseases, University of Modena, Modena, Italy; 4 Microbiology and Virology Laboratory, ASST Papa Giovanni XXIII, Bergamo, Italy

**Keywords:** Dual cART, Dolutegravir, Lamivudine, Switch, Simplification, Costs, Cohort

## Abstract

**Background:**

Little is known about the applicability of dual treatments based on integrase inhibitors. We explored the combination of lamivudine + dolutegravir as an option when switching from standard cART in virologically suppressed patients.

**Methods:**

In this prospective cohort we enrolled patients previously switched to 3TC + DTG who were 18 years or older, with no previous resistance mutations to the used drugs, having a HIV-RNA <50 copies/ml for 6 months or longer, negative for HBsAg and on a stable (>6 months) cART.

**Results:**

Ninety-four individuals were included. They were mostly men (77.7%) with a mean age of 53 years. They presented 159 co-morbidities including cardiovascular, bone, hepatic, kidney, and CNS diseases. Because of these pathologies, they took 207 non-ARV drugs (mean 2.2 per patient). Median duration of viral suppression was 77.5 months (IQR 61). All subjects were prospectively followed up to week 24 and all remained on dual therapy during the whole period. Neither virological failure, nor viral blip was detected.

The median CD4 count rose from 658 cells/mcl (IQR 403) to 724 cells/mcl (IQR 401) (*P* = 0.006) without a significant (*P* = 0.44) change in the CD4/CD8 ratio. A significant (*P* < 0.0001) increment of median creatinine from 0.87 mg/dl (IQR 0.34) to 0.95 mg/dl (IQR 0.29) was observed in the first 2 months but thereafter leveled on these values (1.00 mg/dl; IQR 0.35) (*P* = 0.111 compared to 2 months). The lipid profile slightly improved. The daily cost of cART was significantly (*P* < 0.0001) reduced of 6.89 euros (SD 6.10).

**Discussion:**

Switching to a dual cART regimen based on lamivudine + dolutegravir maintains virological efficacy up to week 24, and is associated to slight improvements of the immunologic and metabolic status. The strategy allows to freely using concomitant medications for associated pathologies. The dual therapy is less expensive in economic terms.

**Conclusion:**

Although still limited evidence exists, a dolutegravir-based dual therapy in combination with lamivudine shows promising results to be confirmed in larger controlled trials.

**Electronic supplementary material:**

The online version of this article (doi:10.1186/s12879-017-2311-2) contains supplementary material, which is available to authorized users.

## Background

In the first years of HIV epidemic the sequential use of nucleoside reverse transcriptase inhibitors (NRTI) as monotherapy or dual therapies rapidly led to treatment failure because of the emergence of resistance-associated mutations [1]. Later, the use of combination antiretroviral therapy (cART), in which two NRTIs were combined with a third agent from a different therapeutic class, became the standard of care. Current treatment guidelines continue the convention of preferred cART based on combining a dual NRTI backbone with a third “anchor” agent [2, 3] as initial treatment. With the improved potency and tolerability and the higher barrier to the development of resistance of newer drugs interest has re-emerged for ARV-sparing strategies including monotherapy and dual therapies. A reduced drug burden could be of interest as patients with HIV are now living longer with an increasing prevalence of comorbidities associated with natural aging, including renal, cardiovascular, or liver diseases; cognitive decline; metabolic disorders such as diabetes and dyslipidemia; and osteoporosis [4]. Drug-related adverse events (AEs) associated with the long-term use of cART may contribute to these comorbidities [5–11].

Dual regimens have been applied as initial therapy in ARV-naive patients or as a switch strategy in those patients who have become virologically suppressed on standard regimens [12–17]. Ideally, these regimens should achieve and maintain viral suppression and immunologic control while minimizing short- and long-term AEs, improve adherence and convenience, and reduce drug-drug interactions and costs. The Italian Guidelines for the treatment of HIV-infected adults [18] contain an entire chapter on “optimization” of cART with less-drug-regimens (LDR). It is recognized that reasons leading to the choice of a LDR (dual or mono therapies) include: a) intolerance to the ongoing regimen; b) presence of co-morbidities on which the current regimen could be detrimental: c) prevention of long-term toxicity; d) current regimen not anymore recommended; e) drug-drug interactions; and f) need to improve treatment adherence.

Little is known about the applicability of dual treatments based on integrase inhibitors and a NRTI. We report a prospective, clinical, uncontrolled experience on patients switched, while virologically suppressed, to the combination of dolutegravir plus lamivudine that is considered by Italian Guidelines as optional (CII) [18].

## Methods

We considered for inclusion in this cohort only patients that, at the moment of therapeutic switch, had a HIV-RNA <50 copies/ml for 6 months or longer. All were negative for hepatitis B virus surface antigen, and were on a stable (>6 months) cART generally based on a nucleosidic backbone plus a third anchor agent, or, in a few cases on other complex regimens. Further, only patients with no previous resistance mutations to either integrase inhibitors or lamivudine were selected. Resistance had to be determined by genotypic analysis before the start of cART or afterward in the occasion of viral blips before the current regimen was started. Patients were not included if they had a viral failure following their last genotypic test.

No experimental procedure (e.g. randomization) was applied, and drugs were used according to a considered alternative option in Italian Guidelines. In all patients, the decision to switch therapy was taken on clinical grounds as they presented a clinically relevant reason, either because of concomitant diseases, altered laboratory tests, drug adverse events or risk of drug-to-drug interactions. The drug combination was presented as a possible alternative and discussed individually according to clinical needs. The possibility to use the dual combination was discussed according to available data [40] and to results obtained with similar dual therapies [15, 16]. Alternatives were presented according to the specific clinical situation and comprehended (but were not limited to): a) possible alternative regimens such as for example 3TC + ATV/r or more complex combinations (either with 2 or 3 active agents e.g. INI + PI + MRV or INI + NNRTI + PI); b) the possibility to add therapies to counter-act specific adverse events (e.g. alendronate for osteoporosis or statins for dyslipidemia) with the possible connected adverse events (to stay with the previous example, gastro-intestinal discomfort; myalgia or myopathy); c) the possibility to increase the frequency of clinical controls to closely monitor clinical alterations (e.g. renal insufficiency); d) the possibility to overcome current adverse events by accepting different potential risks (e.g. stopping TDF for osteoporosis and starting ABC with potential CV risk); e) the possibility to change indicated adjunctive therapies because of risk of drug interactions (e.g. PPIs, Flumetasone, statins, amiodarone); f) the possibility to postpone needed but not life-saving treatments (e.g. DAAs for chronic HCV infection). The choice of the dual regimen was therefore a part of the patient/doctor relationship during normal clinical practice. Although patients were followed prospectively, none of them switched therapy after the decision to perform this analysis, consequently the local EC ruled out that no formal ethics approval was required and patients gave their informed consent solely for the use of clinical and laboratory data.

All patients were switched to a dual combination of dolutegravir (50 mg once daily) plus lamivudine (300 mg once daily). The switch was independent from the decision to include the patients in this cohort. Once included, the patients were followed prospectively for at least six months

Patients were followed accordingly to current clinical practice as indicated from Italian guidelines with visits after 2, 4, 6 month and thereafter every 3–4 months. Each visit included a physical examination and blood and urine analysis performed using standard methods. For the only seek of this analysis, the primary endpoint was the virological response, defined as the proportion of patients with HIV viral load below 50 copies per milliliter 24 weeks after the switch.

Several other commonly collected data were used to evaluate possible secondary endpoints. We analyzed safety and tolerability by questioning patients at each visit and by physical examination and laboratory analysis. We evaluated immunological changes in terms of classical CD4+, CD8+ cell/counts variation and we also collected changes in creatinine and blood lipid content as possible markers of drug toxicity.

Data are presented as medians and interquartile range or percentages. Student’s *t*-test for paired samples was employed to identify significant changes in immunological, renal and metabolic functions. We did all statistical analyses using SPSS version 17.

## Results

Ninety-four individuals switched their regimen. All of them remained on lamivudine + dolutegravir dual therapy for the whole 24 weeks period. Patients continued the dual therapy thereafter and the median follow-up of the cohort was of 17.4 months (IQR 6.6) for 128.5 patient/years at the moment this report was written

Patients were mostly men of middle age and of Italian origin. Baseline characteristics are summarized in Table 1.

**Table 1:** Baseline characteristics of the 94 patients

Characteristic	Median or percentage	IQR
Gender (masculine)	77.7%	
Age (years)	52	13
Risk factor for HIV		
Heterosexual contacts	54.3%	
Homosexual contacts	23.4%	
Intravenous drug use	20.2%	
Other	2.1%	
Number of ARV drug lines	3	4
Time on cART (years)	10	12
Time on current cART (months)	37.5	61
Time below detection limit for HIV (months)	77.5	61
Drugs in baseline ARV regimen (patients treated with drug)		
TDF	52.1%	
ABC	36.2%	
AZT	3.2%	
EFV	28.7%	
NVP	13.8%	
RPV	10.6%	
ETR	4.3%	
ATV/r	13.8%	
DRV/r	12.8%	
APV/r	1.1%	
LPV/r	1.1%	
RAL	9.6%	
DTG	5.3%	
EVG	2.1%	
CD4 (cells/mcL)	673	403
Reasons for drug switch		
Concomitant disease	30.9%	
Abnormal laboratory test	28.7%	
Adverse Events	19.1%	
Drug/drug interaction	13.8%	
Concomitant diseases + abnormal laboratory test	4.3%	
Adverse events + drug/drug interaction	2.1%	
Abnormal laboratory test + drug/drug interaction	1.1%	

The patients had a long cART history and were on average on their fourth line of therapy. Most of patients (93.6%) were, at baseline, taking a triple-drug regimen, being the most common backbones tenofovir + emtricitabine (52.1%) or abacavir + lamivudine (36.2%). The most common anchor drugs were efavirenz (28.3%) and nevirapine (13.8%) among NNRTIs and either boosted atazanavir (13.8%) or boosted darunavir (12.8%) among PIs. A previous exposure to INI was documented in 14.9% of individuals. Only 6 subjects were on a dual therapy maraviroc + boosted darunavir (2); raltegravir + boosted darunavir (2); etravirine + boosted darunavir and etravirine + raltegravir (1 each).

**Fig. 1:**
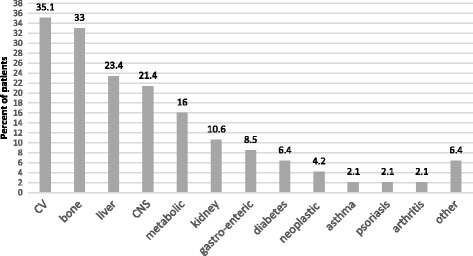
Concomitant diseases in the 94 patients

**Fig. 2:**
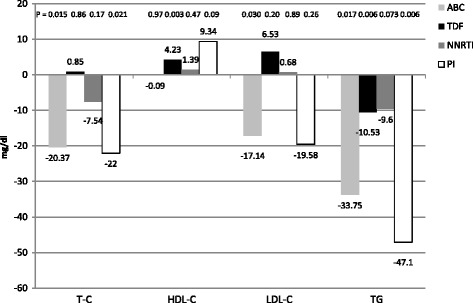
Lipid variation. Difference in blood concentrations between baseline and 6 months

The main reasons for therapeutic switch were concomitant diseases and abnormality of laboratory tests followed by drug related adverse events or possible adverse events or a potential drug-drug interaction. A mix of reasons was present in a remaining minority of patients (Table 1). Overall, they presented 159 co-morbidities (Fig. 1). Because of these pathologies, the patients took 207 different drugs (mean 2.2; SD 1.7 drugs per patient) including, but not limited to, diuretics, beta-blockers, Ca-antagonists, ASA, statins, benzodiazepines, vitamins, PPI, insulin, metformin. At baseline all patients had a viral load < 50 copies/ml. The same HIV-RNA level was detected in all patients after 2 and 6 months from the switch. Neither virological failure, nor viral blip above 50 copies/ml was detected. The median baseline CD4 count was 673 cells/mcl (IQR 403), at six months it raised to 724 cells/mcl (IQR) (P = 0.006) without a significant (*P* = 0.44) change in the CD4/CD8 ratio that varied from 0.83 (IQR 0.75) to 0.95 (IQR 0.77). A significant (*P* < 0.0001) increment of mean creatinine, 0.06 mg/dl in magnitude, was observed in the first two months raising the baseline value of 0.87 mg/dl (IQR 0.34) to 0.95 mg/dl (IQR 0.29), but thereafter leveled on these values being the median after 6 months 1.00 mg/dl (IQR 0.35) (*P* = 0.111 compared to 2 months). The lipid profile slightly changed after switching to the dual regimen: total cholesterol −7 mg/dl (*p* = 0.047); LDL-cholesterol −7 mg/dl (*P* = 0.355); HDL-cholesterol +4 mg/dl (*P* = 0.036) and triglycerides −31 mg/dl (*P* = 0.012); although differences were dependent on pre-switch type of cART, too (Fig. 2).

During the 6 month follow-up 3 patients were admitted to hospital because of causes judged unrelated to cART: diabetes, sub-dural hematoma and coronary stent positioning (one each).

At the end of follow-up (median 17.4 months) all patients still receiving the same ARV therapy (91/94) had a viral load was < 50 copies/ml and their median CD4 count was 763 cells/mcl (IQR 491) (*P* = 0.002 vs baseline). One patient was lost to follow-up and two patients died between the sixth month of therapy and the end of follow-up because of variceal bleeding due to an alcoholic cirrhosis and because of pulmonary cancer with brain metastasis (one case each). Finally, by changing regimen, the daily cost of cART was significantly (*P* < 0.0001) reduced of 6.89 euros (SD 6.10 euros) (Additional file 1).

## Discussion

Toxicities associated with antiretroviral agents are often drug class specific. Hyperlipidemia has been commonly associated with protease inhibitor (PI) use [19, 20], whereas mitochondrial toxicity (lipoathrophy, functional kidney dysfunction, cardiovascular accidents, and osteoporosis) [21, 22] has been associated with nucleoside analogs use. To overcome this situation, different strategies considering PI/rtv-based dual regimens have been proposed. Most of them addressed the association of raltegravir [12, 13, 23]; lamivudine [14–16]; a non-nucleoside inhibitors (NNRTI) [17, 24] or maraviroc [25] to the boosted PI.

So far, however, the need of maintaining the efficacy and convenience of robust cART forced clinicians to include a boosted-PI in dual-therapy regimens. Consequently, the potential benefits for some organ systems (e.g., kidney, cardiovascular disease, and bone) were balanced by potential detriments in others (e.g., hyperlipemia).

The integrase inhibitor (INI) class has been increasingly recognized as a first-line option, especially because of its good efficacy and tolerability [2, 3].

In combination with two NRTI, the INI class was found to be superior to boosted-PIs [26, 27] or efavirenz [28–30] in large controlled studies, even though raltegravir showed a lower genetic barrier compared to dolutegravir [26].

In fact, dolutegravir is not only as efficient as raltegravir as a first-line strategy, but, similarly to boosted-darunavir, it is characterized by a high affinity to its target, resulting in strong and sustained binding [29]. Therefore, in vitro selection of mutants resistant to dolutegravir is very difficult.

To date, no emergent dolutegravir-resistant virus has ever been reported in a patient in whom dolutegravir was prescribed as a first therapy [31]. Nevertheless, patients in whom a first-generation INI has failed may have selected a pathway leading to cross-resistance, including dolutegravir [32]. As a matter of fact, a few recent communications have explored the possibility of dolutegravir monotherapy [33–36] and two of them reported 4 patients out of 61 (6.6%) who failed while on dolutegravir as monotherapy: all of them had been exposed to a first-generation INI (i.e. raltegravir or elvitegravir) [33, 34]. Better results have been obtained in some studies exploring the switch to a dual therapy using dolutegravir either in combination with rilpivirine [36, 37] or lamivudine [36, 38, 39], the latest being explored in naïve patients, too [40, 41]. Although small in numbers and heterogeneous in nature, these experiences have documented a substantial virological efficacy and tolerability of the dual regimens without exposing patients to the risk of selecting for INSTI-inducing resistance mutations.

To our knowledge, we report the largest cohort of patients simplified to the dual 3TC + dolutegravir regimen. The studied cART was effective in maintaining HIV-RNA suppression in a cohort of treatment-experienced participants. We did not observe any virological failure, or any viral blip over the 50 copies/ml threshold. The switch therapy offered hints of an improved immunological outcome even in patients who already immune-reconstituted. The reduction of mitochondrial toxicity due to the reduced utilization of NRTIs may possibly explain this result [42]. Therapy was well tolerated and no patients stopped therapy because of low tolerability of dolutegravir. This is in contrast with the results recently described by a Dutch group [43] suggesting a high proportion of psychiatric adverse events leading to dolutegravir discontinuation, despite the fact that our population, although numerically smaller, was wide enough to detect a problem of that size. Being all our patients treatment-experienced may have affected their perception of the overall tolerability of the regimen. Alternatively, the use of a reduced number of active drugs and/or the limitation of NRTI use may improve treatment acceptance. In our study, the dual combination resulted clinically neutral on the renal function and the creatinine increment we observed, although statistically significant, was of limited entity, occurred soon after the switch and stabilized thereafter. These changes are consistent with dolutegravir action as an inhibitor of the renal protein organic cation transporter 2 (OCT2) [44, 45].

As far as the lipid profile is concerned, our results are substantially similar to previously reported findings using the same drug combination [39]. We observed a reduction of triglycerides and total cholesterol, an increment of HDL-cholesterol, and stable levels of LDL-cholesterol; however, the pre-switch therapies influencing the baseline lipidic asset may influence the post-switch variation entity. With this respect, the use of triple drug combinations including boosted-PIs or backbones including abacavir may explain differences with previous experiences [36, 39].

Along with treatment-emergent toxicities, concomitant pathologies and the potential interactions with drugs used to treat these diseases were among the most frequent reasons for switching to the dual drug regimen. With this respect the combination of lamivudine and dolutegravir was safe and did not limit therapeutic choices (e.g. statins, GI-tract drugs, etc.). Finally, being the generic form of lamivudine available, the dual combination was economically convenient compared to all preferred regimens [2, 3].

Clearly, this observational study has several limitations: first of all, its nature does not allow for comparison with a control group, although we tried to limit analysis bias with the prospective design; second, the limited (in number) inclusion criteria allowed for a rather heterogeneous population (i.e.: wide CD4+ T-cell range, reasons for switching, associated drugs). We tried to limit this confounding bias by enrolling into the cohort only patients with a known therapeutic history and a controlled HIV viremia. Finally, besides maintenance of virological suppression, we analyzed only a small group of safety parameters and considered as relevant only adverse events leading to treatment discontinuation. This may be a limit in a research setting, but strictly reflects a clinical relevance.

## Conclusion

In conclusion, we demonstrated in an uncontrolled cohort of pre-treated individuals that switching to a dual cART regimen based on the association of lamivudine and dolutegravir is virologically effective up to 24 weeks, and is associated to slight improvements of the immunologic and metabolic status. This regimen should still be considered as under-investigated and its use cannot be considered routinely. However, the strategy, compared to several alternative ARV regimens, allows to reduce the risk of drug-drug interactions and to use safely concomitant medications for associated pathologies. The dual therapy, at least in our economic environment, is less expensive than most alternative ARV regimens, too.

In the near future, with the availability of new nucleotidic molecules [44] the clinical concern about some of the variables that lead to the therapeutic switch (NRTI-related toxicities) could become less compelling, however, having therapeutic alternatives is always to be considered advantageous as can allow to personalize therapy in individual patients. Although still limited evidence exists, a dolutegravir-based dual therapy in combination with lamivudine shows promising results to be confirmed in larger controlled trials.

## Additional file

Additional file 1: DTG-3TC Anonymous db for paperR1. (XLSX 82.1 kb)

## Abbreviations

95%CI: 95% confidence interval
AEs: Drug-related adverse events
cART: Combination antiretroviral therapy
INI: Integrase inhibitor
NNRTI: Non nucleoside reverse transcriptase inhibitors
NRTI: Nucleoside reverse transcriptase inhibitors
PI: Protease inhibitor
RTV: Ritonavir
